# Bioassay-Guided Isolation and HPLC Determination of Bioactive Compound That Relate to the Antiplatelet Activity (Adhesion, Secretion, and Aggregation) from *Solanum lycopersicum*

**DOI:** 10.1155/2012/147031

**Published:** 2012-11-13

**Authors:** Eduardo Fuentes, Ricardo Castro, Luis Astudillo, Gilda Carrasco, Marcelo Alarcón, Margarita Gutiérrez, Iván Palomo

**Affiliations:** ^1^Immunology and Haematology Laboratory, Immunohaematology and Clinical Biochemistry Department, Faculty of Health Sciences, Universidad de Talca, 3460000 Talca, Chile; ^2^Centro de Estudios en Alimentos Procesados (CEAP), CONICYT-Regional, Gore Maule, R09I2001 Talca, Chile; ^3^Synthesis Laboratory, Chemical Institute of Natural Resources, Universidad de Talca, 3460000 Talca, Chile; ^4^Horticulture Department, Faculty of Agricultural Sciences, Universidad de Talca, 3460000 Talca, Chile

## Abstract

In seeking the functionality of foodstuff applicable to medicine, ripe tomato fruits were found to show an antiplatelet activity. Therefore, the bioactive compound was isolated, structurally identified, and studied for an inhibitory effects on platelet adhesion, secretion, and aggregation. The concentration of adenosine in ripe tomato fruits (pulp and skin extracts) and its processing by-products (paste and pomace) was determined by reversed-phase high-performance liquid chromatography (HPLC). According to platelet aggregation inhibition induced by ADP, the total extract residual was fractionated by liquid-liquid separation, obtaining aqueous, ethyl acetate and petroleum ether extracts. The aqueous extract was subjected to repeated permeation over sephadex LH-20 and semipreparative TLC. The isolate finally obtained was identified as adenosine on the basis of ESI-MS, ^1^H NMR, HPLC, and UV spectra. Adenosine concentration dependently (2.3–457 **μ**M) platelet aggregation inhibited induced by ADP. Also, adenosine present inhibition of platelet secretion and thrombus formation under flow conditions. The quantitative HPLC analysis revealed significant amounts of adenosine in ripe tomato fruits and its processing by-products. From these results, extracts/fractions of ripe tomato fruits and their processing by-products may be referred to as functional food and functional ingredients containing a compound that inhibits platelet function with a potent preventive effect on thrombus formation, as those that occur in stroke.

## 1. Introduction

According to the World Health Organization, cardiovascular disease (CVD) (i.e., acute myocardial infarction, cerebrovascular disease, and peripheral arterial thrombosis) represents about 30% of deaths worldwide [1], with a relative increase over time due to the aging of the population [2]. 

The current lifestyle of the population contributes to the development of risk factors for CVD, such as hypertension, diabetes, smoking, and hypercholesterolemia [3, 4]. The development and progression of CVD lie in the interactive processes of atherosclerotic lesions and thrombus formation, an interaction established primarily by platelet participation [5].

In the context of atherosclerosis, platelets can adhere to endothelial cells and contribute to the recruitment of leukocytes involved in the local vascular inflammation [6, 7]. Platelets also intensify the inflammatory process at all stages of atherosclerosis by expressing membrane molecules such as intercellular adhesion molecule-2, P-selectin, and CD40L [8, 9]. Also, when there is a damaged atheromatous plaque, platelets adhere, secrete their contents, and then aggregate to it [10]. This activation is a dynamic process that can lead to intermittent or permanent obstruction to blood flow, resulting in ischemic tissue injury and organ dysfunction [11].

From the point of view of public health, efforts should be directed to primary prevention, namely, to reduce cardiovascular risk factors mentioned above [12, 13]. In this context, regular consumption of fruits and vegetables, apart of the called Mediterranean diet [14], might be related to the bioactive compounds found in them [15], which explains the increasing amount of attention in research on phytochemicals in the prevention of CVD [16]. Tomatoes (*Solanum lycopersicum*), fresh or processed (e.g., tomato paste), apart from their nutritional value, have been found to provide a cardioprotective effect, both at the endothelial and platelet levels [17].

The purpose of this paper was to isolate and identify one of the bioactive compounds of tomatoes that present antiplatelet activity (adhesion, secretion, and aggregation) and also to examine extensively the effect of different kinds of tomato processing, in the production of paste and its by-product pomace on the content of antiplatelet compound.

## 2. Materials and Methods

### 2.1. Chemicals and Reagents

Acetonitrile HPLC grade from Merck (Darmstadt, Germany) was used as well as Sephadex LH-20 (Pharmacia Fine Chemicals, Piscataway, NJ, USA) and thin layer chromatography (TLC) plates (Merck, Darmstadt, Germany), whereas acetylsalicylic acid, 1,1-diphenyl-2-pycrylhydrazyl (DPPH), quercetin, catechin, and adenosine were purchased in Sigma-Aldrich (St. Louis, Missouri/MO, USA). Sodium chloride (p.a.), potassium chloride (p.a.), sodium phosphate dibasic (p.a.), potassium phosphate monobasic (p.a.), petroleum ether, methanol, and ethyl acetate were obtained from Arquimed (Santiago, Chile). The agonist adenosine 5′-diphosphatebis (ADP), calcein-AM, collagen, and bovine serum albumin (BSA) were obtained in Sigma-Aldrich (St. Louis, Missouri/MO, U.S.A), whereas luciferase luciferin reagent was obtained from Chrono-Log corp. (Havertown, PA) and microfluidic chambers were from Bioflux (Fluxion, San Francisco, California, USA).

### 2.2. Processing Material

Processing tomato (H9665, H9997, and H7709), pomace and tomato paste were obtained from “Tresmontes Luchetti” (Production plant Talca, Chile). 

### 2.3. Industrial Quality Measurement

The industrial quality through firmness expressed in pounds (McCormick Fruit Pressure Tester model FT 327, Yakima, Wash) and soluble solids expressed in °Brix (Meiji-La Bax HT 0-32) were determined from a sample of harvested fruits (10/experimental unit).

### 2.4. Approximate Chemical Composition

To characterize the chemical composition of the pulp of tomatoes, the water content (%) was determined by drying in a convection oven at 60°C, the protein content was measured using the Kjeldahl method, and fat content was determined by Soxhlet method. Ash content was obtained by drying the samples at 550° in a muffle furnace C for two hours. Crude fiber was determined by the acid sequence method using 1.25% H_2_SO_4_ and 1.25% NaOH for acid and alkaline hydrolysis, respectively. Carbohydrate content was calculated as the difference between the total and the contents of all other ingredients [18]. Each measurement was performed in triplicate.

### 2.5. Extraction and Fractionation

Tomatoes were carefully washed; skin and seeds were manually separated from the pulp. Then the pulp was homogenized in a blender (Somela BL1500) and extracted three times with MeOH (3 × 15 L each) at room temperature in the dark for 12 h per extraction. The mixture was sonicated (Elma Transsonic 700/H, Singen, Germany) for 5 min and then filtered through gauze twice. The filtrate was evaporated under *vacuo* (Laborata 4001, Heidolph, Germany or RE 111-B461, Buchi Labortechnik, The Netherlands) to remove methanol. The total extract residuary was fractionated by liquid-liquid separation, obtaining petroleum ether, ethyl acetate, and aqueous extracts. Since the aqueous extract had significant antiplatelet properties further purification was carried out. The aqueous extract was lyophilized (Freezone 6, Kansas City, Missouri, Labconco, USA).

#### 2.5.1. Isolation

The aqueous extract was subjected to a repeated permeation over Sephadex LH-20 (column length 60 cm, internal diameter 3 cm) using MeOH : H_2_O 4 : 1 as eluent. The fractions were monitored at 254 nm using a He*λ*ios R V-3.06 spectrophotometer (Unicam spectrometry, Cambridge, UK) and analyzed by HPLC (A: H_2_O and B: acetonitrile; 0–10 min linear gradient 98–2% B, and 10–20 min linear gradient 95–5% B, 20–30 min linear gradient 50-50% B, and finally, washing the column with 100% B for 10 min). 

Semipreparative TLC was performed on 10 cm × 20 cm TLC silica gel plates coated with 1 mm layer and the sample was applied. The plate was developed using 25 mL of mobile phase EtOAc : AcOH : H_2_O 10 : 2 : 3 v/v/v in a saturated chamber. The plate under UV light (254 nm) was developed. The bands observed were removed, extracted with methanol, and concentrated. Since one of the bands had significant antiplatelet properties, further identification was carried out.

### 2.6. Identification of Antiplatelet Compound

#### 2.6.1. Spectral Scanning

Spectral scanning between 200 and 600 nm was used to investigate the UV-visible maximum band absorption. The UV/Vis spectra were obtained in a spectrophotometer using MeOH as a solvent.

#### 2.6.2. NMR Analysis

The structure of band was determined by ^1^H NMR on a Bruker AMX spectrometer (Bruker, Germany) operating at 400 MHz, using DMSO-D_6_ as a solvent. TMS was used as an internal standard. Chemical shifts (*δ*) and *J* values were reported in ppm and Hz, respectively, relative to the solvent peak (DMSO-D_6_ at 2.50 and 3.34 ppm for protons). Signals were designated as follows: s, singlet; d, doublet; dd, doublet of doublets; t, triplet; m, multiplet.

#### 2.6.3. Mass Spectrometer

ESI-MS/MS data was collected using a high resolution hybrid quadrupole (Q) and orthogonal time-of-flight (TOF) mass spectrometer (Micromass Q-Tof, UK) with constant nebulizer temperature of 80°C. The ESI source and the mass spectrometer were operated in a negative ion mode, and the cone and extractor potentials ware of 10 eV, with a scan range of *m/z* 100–500. The band infused into the ESI source at flow rates of 5 *μ*L min^−1^ was dissolved in acetonitrile ion-induced dissociation (CID) with argon in the collision chamber. The values expressed are average mass and correspond to the [M-H]^−^ ion.

### 2.7. Preparation of the Standard Curve and Extracts for Quantitative Analysis

Standard solutions of adenosine 3.75 mg/mL for quantification were prepared dissolving 187.5 mg in a volumetric flask of 50 mL (PBS: phosphate buffered saline pH 7.4, 0.1 M). The points of the curve were realized by the dilution of the stock solution between 3.75 and 0.19 mg/mL to produce a sequence of 3.75, 1.88, 0.75 and 0.19 mg/mL in buffer PBS. Extracts of ripe tomato fruits (pulp and skins extracts) and their by-products of processing (paste and pomace extracts) were lyophilized [19] and then equilibrated to room temperature for 1 h and dissolved in 1000 *μ*L of PBS. The content of adenosine was expressed in mg adenosine/mg dried extract. All samples were filtered through a Millex-LS PTFE filter with 5 *μ*m pore size (Millipore Corporation, Billerica, MA, USA); before HPLC analysis, the injection was made in duplicate for 100 *μ*L.

### 2.8. HPLC

The quantitative study of the adenosine in ripe tomato fruits and its by-products of processing extracts were performed by HPLC. The HPLC system (Agilent ChemStation, 1200, USA) consisted of a low-pressure quaternary pump (model Agilent 1200) and autosampler (model Agilent 1260 Infinity Autosampler) with 99-vial capacity sample. Separations were carried out on a LiChrospher RP-18 column of 250 mm (5 *μ*m) particle size. A guard column (LiChrospherRP) select B (5 *μ*m) particle size was placed in front of the analytical column. The chromatographic conditions were the following: PBS mobile phases which were subjected to filters (5.0 *μ*m) and gradient programmed isocratic, room temperature, run time 23 minutes, injection volumes 100 *μ*L, and wavelengths 254 [20].

### 2.9. Antioxidant Activity

#### 2.9.1. DPPH Free Radical Scavenging Assay

The scavenging activity of the extracts was estimated using DPPH as the free radical model according to the method adapted from Molyneux [21]. An aliquot of 750 *μ*L of samples and control (80% methanol) were mixed, respectively, with 1.5 *μ*L of DPPH for final concentration of 100, 500, and 1000 *μ*g/mL. The mixture was shaken vigorously and left to stand at room temperature for 30 min in the dark. The mixture was measured spectrophotometrically at 515 nm. The free radical scavenging activity was calculated as percentage of DPPH discoloration using the following equation (1): percentage of scavenging DPPH free radical = 100 × (1 – AE/AD), where AE is the absorbance of the solution after adding the extract at a particular level, and AD is the absorbance of the blank DPPH solution. Quercetin and catechin were used as reference compounds. Each measurement was performed in triplicate.

### 2.10. Effect on Platelet Function

#### 2.10.1. Preparation of Human Platelet Suspensions

Venous blood samples were taken from two volunteers (healthy university students), who previously signed informed consent in 3.2% citrate tubes (9 : 1 v/v) by phlebotomy with vacuum tube system (Becton Dickinson Vacutainer Systems, Franklin Lakes, NJ, USA). The protocol was authorized by the Ethic Committee of Universidad de Talca in accordance with the Declaration of Helsinki (approved by the 18th World Medical Assembly in Helsinki, Finland, 1964). The samples were gently homogenized by 5-fold inversion and allowed to stand for 5 minutes. Then they were centrifuged (DCS-16 Centrifugal Presvac RV) at 240 g for 10 minutes, and 1 mL of platelet-rich plasma (PRP) was taken from each tube for platelet count (in triplicate) in an hematologic counter (Bayer Advia 60 Hematology System, Tarrytown, NY, USA). The original tubes were centrifuged at 650 g for 10 minutes to obtain the platelet-depleted plasma (PDP). Finally, the PRP was adjusted to 2 × 10^5^ platelets/*μ*L with PDP. 

#### 2.10.2. Platelet Antiaggregating Activity

Platelet aggregation was monitored by light transmission turbidimetric method according to Born and Cross [22], using a lumiaggregometer (Chrono-Log, Havertown, PA, USA). Briefly, 480 *μ*L of PRP in the reaction vessel were preincubated with 20 *μ*L of sample, negative control (saline 0.9%), or positive control (acetylsalicylic acid 110 *μ*M). After 3 min of incubation, 20 *μ*L of agonist was added to initiate platelet aggregation, which was measured for 6 min. ADP 8 *μ*M was used as an agonist. All measurements were performed in triplicate. The results of platelet aggregation (maximum aggregation (%), slope, area under and lag time (s)) were determined by the software AGGRO/LINK (Chrono-Log, Havertown, PA, USA) and the relative inhibition of the maximum platelet aggregation: 100 − ((%AgX ∗ 100)/%AgC) (% AgX: relative aggregation of the component under study, % AgC: relative control aggregation).

#### 2.10.3. Platelet Secretion Assay

Platelet secretion was determined by measuring the release of ATP using luciferin/luciferase reagent. Luciferin/luciferase (50 *μ*L) was added to 480 *μ*L of platelet suspension (PRP adjusted to 2 × 10^5^ platelets/*μ*L) within 2 min before stimulation. Platelet secretion was recorded in real time in a lumiaggregometer at 37°C with stirring (1000 rpm) and luminescence (×0.2). To examine the effects on platelet secretion, platelets were preincubated with aqueous extract and adenosine for 2 min prior to the addition of ADP 8 *μ*M [23].

#### 2.10.4. Analysis of Platelet Adhesion and Thrombus Formation in Flowing Whole Blood

For flow experiments, BioFlux 200 flow system (Fluxion, San Francisco, California, USA) with high shear plates (48 wells, 0–20 dyne/cm^2^) was used. Using manual mode in the BioFlux software, the microfluidic chambers were coated for 1 hour with 50 *μ*L of collagen 200 *μ*g/mL at a wall shear rate of 200 s^−1^. 

The plaque coating was allowed to dry at room temperature for one hour. The channels were perfused with PBS for 10 min at a wall shear rate of 200 s^−1^ for removing the interface. Then, the channels were blocked with BSA 0.5% for 10 min at a wall shear rate of 200 s^−1^. In order to visualize platelets, the citrate-anticoagulated blood containing calcein-AM (4 *μ*M) was added to the inlet well, and chambers were perfused for 10 min at a wall shear rate of 1000 s^−1^.

The plaque-coated microfluidic high shear plates were mounted on the stage of an inverted fluorescence microscope (TE200, NIKON, Japan). Control blood (saline 0.9%) and blood with aqueous extract (1 mg/mL) or adenosine (114 *μ*M) were preincubated at room temperature for 1 hour prior to the start of flow, and experiments were performed at room temperature [24].

Platelet deposition was observed and recorded in real time (30 frames per min) with a CCD camera (QICAM, QIMaging, Surrey, BC, Canada). We used bright field and fluorescence microscopy for real-time visualization of platelet adhesion and aggregation in flowing blood. For each flow experiment, perfused surface fields of the size of 237900 *μ*m^2^ (located in the middle of the channels of the viewing window) were recorded, and fluorescence images were later analyzed offstage by quantifying the area covered by platelets with the Image J software (version 1.26t, NIH, USA). In each field, the areas covered by platelets were quantified.

### 2.11. Statistical Analysis 

Mean ± standard deviations (SD) were determined using SPSS version 17.0. The data was statistically analyzed by Student's paired or unpaired *t*-test. A Pearson correlation test was used to evaluate the correlation between the adenosine content and inhibition platelet aggregation. The statistical significance level was set up at *P* < 0.05.

## 3. Results

### 3.1. Industrial Quality Measurement

Processing tomatoes presented firmness of 5.2 ± 0.2 pounds and soluble solid content of 5.5 ± 0.8° Brix, thus a ripening stage VI (red), according to the method proposed by Dumas et al.(2003) [25].

### 3.2. Characteristics of Tomato Pulp

The approximate chemical composition of tomato pulp showed moisture 94 ± 2%, protein 12 ± 0.1%, fat 3 ± 0.1%, ash 15 ± 0.1%, carbohydrate 63 ± 0.4%, and crude fiber 7 ± 0.2%, values in concordance with Fuentes et al. [19]. 

Eighteen grams (18 g) (0.3% w/w yield) of a yellow aqueous extract were obtained from 6 kg of tomato pulp. Such extract showed the highest yield over ethyl acetate extract (0.05% w/w yield) and petroleum ether extract (1.4 × 10^−3^% w/w  yield).

When comparing different types of extracts, differences in their antioxidant potential were significant. At a concentration of 1000 *μ*g/mL, ethyl acetate extract (87 ± 2%) was superior to ether petroleum (6 ± 2%, *P* < 0.05) and aqueous extract (15 ± 5%, *P* < 0.05).

### 3.3. Bioassay-Guided Isolation of Antiplatelet Compound

**Figure 1:**
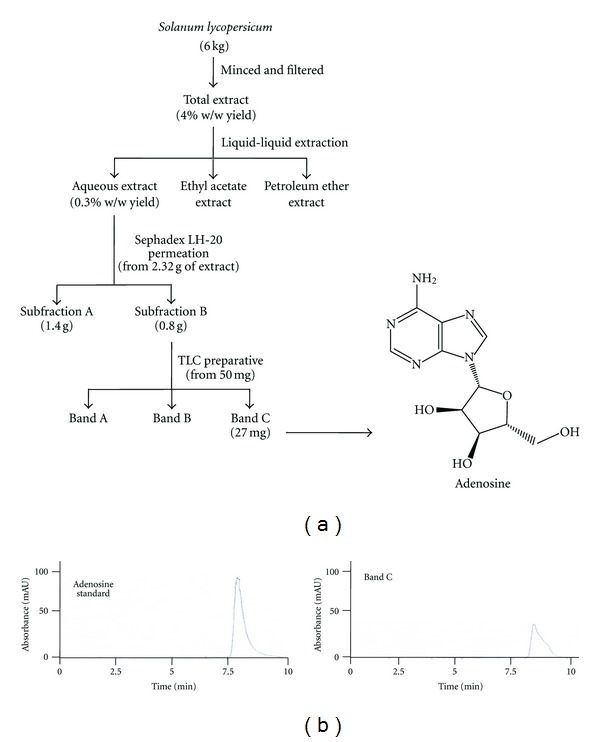
Biodirected isolation and identification of adenosine from *S. lycopersicum.* (a) Extraction and fractionation of pulp from *S. lycopersicum* and (b) chromatograms of adenosine standard and Band C dissolved in PBS.

To advance in the search for a bioactive compound with antiplatelet activity, the aqueous extract was subjected to repeated permeation over Sephadex LH-20 using MeOH : H_2_O 4 : 1 as eluent and 21 fractions of 17 mL each were collected. The fractions were monitored at 254 nm and two subfractions were identified by HPLC (subfractions A and B). Since platelet aggregation induced by ADP was completely inhibited by subfraction B at 1 mg/mL further purification was carried out (Figure 1(a)). 

The subfraction B (50 *μ*g by plate) was applied on semipreparative TLC. Under UV light (254 nm) three bands (BA, BB, and BC) were observed and removed, and extracted with methanol. Then each band was filtered and evaporated under *vacuo*. Since platelet aggregation induced by ADP was completely inhibited by BC at 1 mg/mL, further identification of compound was carried out. 

### 3.4. Identification of the Antiplatelet Compound

The BC band was identified as adenosine according to the UV spectrum (*λ*_max⁡_ = 221 and 261 nm); it had an [M-H]^−^ at *m*/*z* 266.7856 [26] and a retention, time similar at adenosine standard (*R*_*t*_ = 7.8 min by HPLC) (Figure 1(b)). The structure was confirmed by NMR spectroscopy. The ^1^H NMR spectrum of BC was consistent with the structure of adenosine *δ* (ppm): 8.347 (1H, s); 8.133 (1H, s); 7.353 (2H, s); 5.870 (1H, d, *J* = 6.36 Hz); 5.44 (2H, m); 5.15 (1H, m); 4.610 (1H, m, *J* = 6.00, 11.40 Hz); 4.136 (1H, m); 3.960 (1H, m); 3.365 (1H, m); 3.549 (1H, m); for C_10_H_13_N_5_O_4_ found following is obtained: C: 44.94 H: 4.90 N: 26.21 and O: 23.95. The data obtained was consistent with previous reports [27].

### 3.5. Effect of Aqueous Extract and Adenosine on Platelet Secretion and Aggregation

The results of platelet aggregation induced by the agonist ADP with extract, fraction, and adenosine (band C) are presented in Table 1. After the liquid-liquid separation, the inhibition of platelet aggregation induced by ADP in aqueous extract increased to 54 ± 8% (*P* < 0.05). As well as inhibiting the platelet aggregation, the aqueous extract inhibited the platelet secretion in 50 ± 5%.

**Figure 2:**
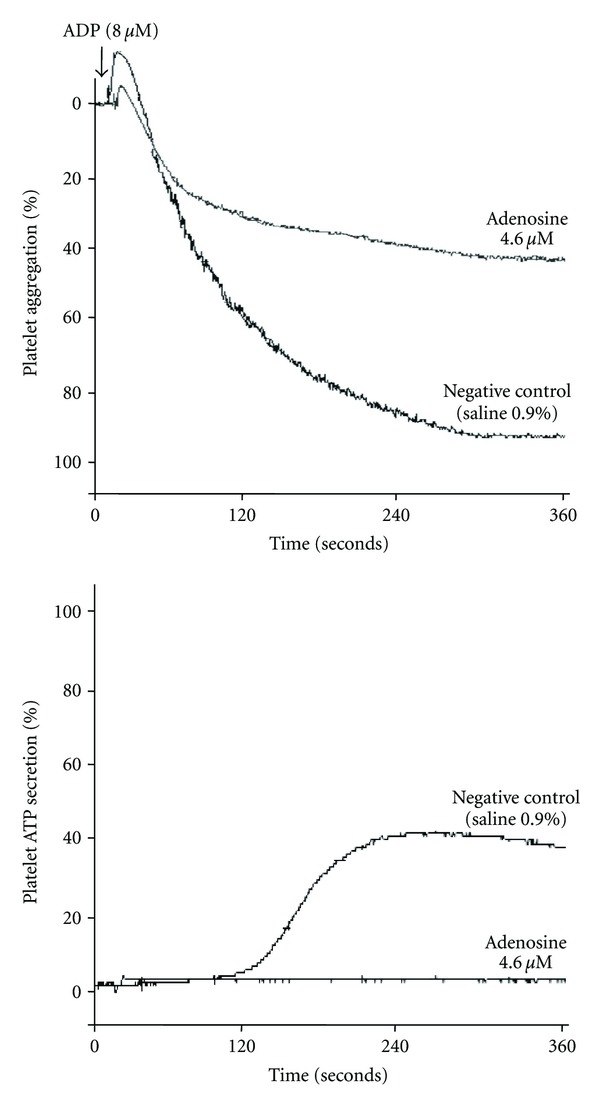
Adenosine 4.6 *μ*M inhibited platelet aggregation and secretion. Luciferin/luciferase reagent and then ADP 8 *μ*M were added to platelets to induce aggregation and secretion, which were recorded in real time using the lumiaggregometer.

The inhibited ADP-induced platelet aggregation of adenosine was concentration dependent (2.3–457 *μ*M), in which a concentration of 4.6 *μ*M inhibited 50 ± 12% platelet aggregation (*P* < 0.05). At the same concentration, it completely inhibited platelet secretion (Figure 2). Besides inhibiting platelet aggregation, adenosine 457 *μ*M displayed a net lag time of 53 ± 2 s (*P* < 0.05).

### 3.6. Aqueous Extract and Adenosine Inhibit Collagen-Induced Platelet Thrombus Formation under Flow Conditions

**Figure 3:**
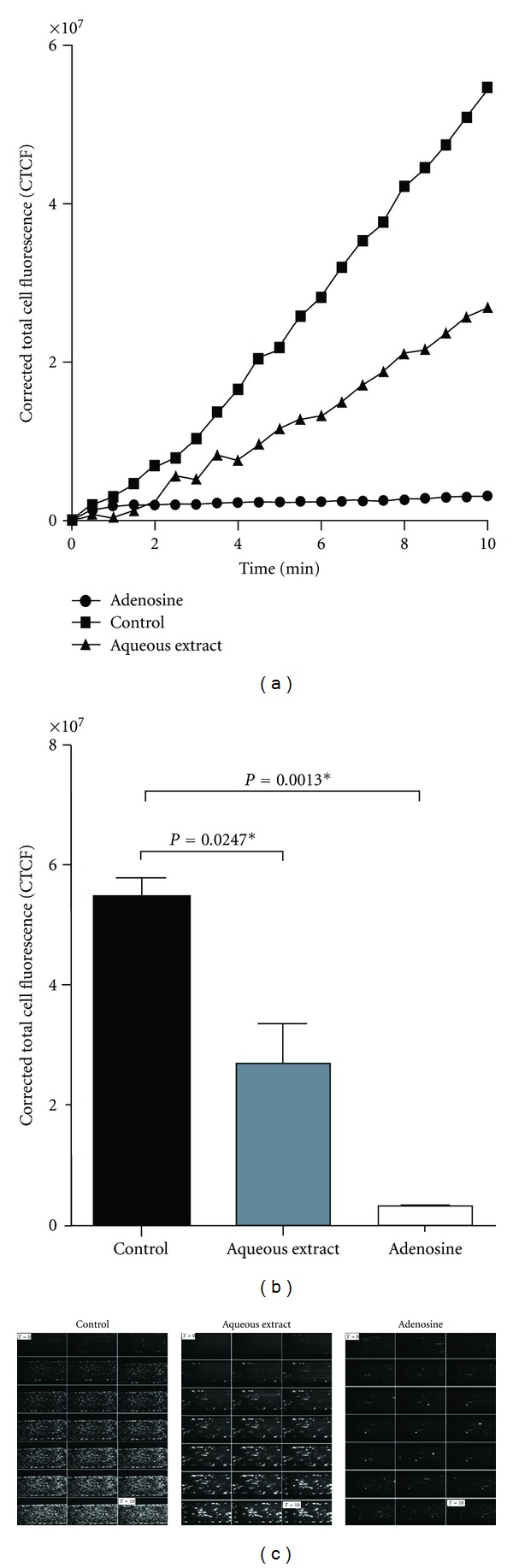
Effect of aqueous extract and adenosine on collagen-induced platelet thrombus formation under arterial flow conditions. Citrate-anticoagulated blood was preincubated with aqueous extract (1 mg/mL), adenosine (114 *μ*M), or negative control (saline 0.9%) for 1 hour and then was perfused over plaque-coated surfaces for 10 min at room temperature at a shear rate of 1000 s^−1^. (a) It shows the intensity (CTCF) over a time lapse, (b) bar diagram (values are mean ± SD; *n* = 3), and (c) time lapse of 10 min at 1000 s^−1^, at 30 sec intervals. *P < 0.05.

The effects of aqueous extract and adenosine on human collagen-induced platelet aggregation and thrombus formation under arterial flow conditions are shown in Figure 3. After perfusion of citrate-anticoagulated blood over plaque-coated surfaces with collagen at 37°C with a wall shear rate of 1000 s^−1^ for 10 min, rapid platelet adhesion and aggregate formation were observed (additional file Movie C1; Figure 3). 

Aqueous extract and adenosine reduced collagen-induced platelet adhesion and aggregate formation under controlled flow. After aqueous extract incubation of blood, the platelet coverage was inhibited by 51.6 ± 16% (*P* < 0.05) (additional file Movie P1; Figure 3). For adenosine, the inhibition of platelet adhesion and aggregate formation under controlled flow were concentration dependent (data not shown), in which a concentration of 114 *μ*M was inhibited by 94.5 ± 10% (*P* < 0.05, compared with the negative control) the platelet coverage (additional file Movie A1; Figure 3). 

### 3.7. Platelet Antiaggregating Activity in Ripe Tomato Fruits and Its by-Products of Processing

**Figure 4:**
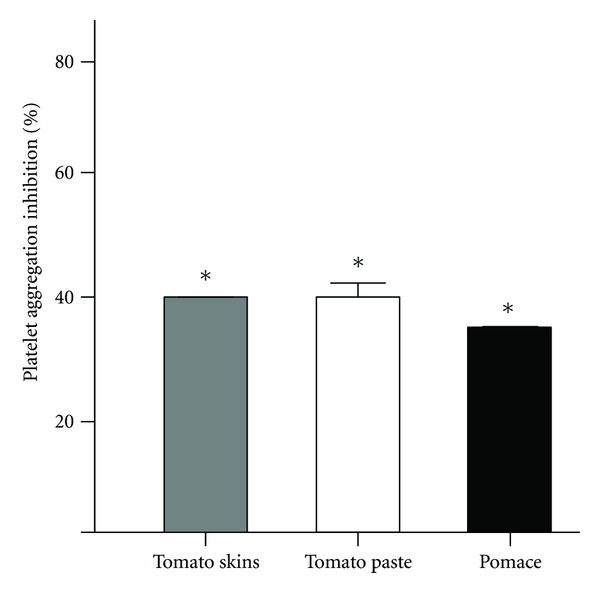
Effect of ripe tomato fruits and their by-products of processing on platelet aggregation. ADP 8 *μ*M. Extracts at 1 mg/mL. *P < 0.05 versus negative control (saline 0.9%).

The inhibition of platelet aggregation ADP-induced by the methanol extract of tomato skins (1 mg/mL) was 40 ± 1% as compared to control (*P* < 0.05). Finally, the aqueous extracts (1 mg/mL) of tomato paste and pomace inhibited platelet aggregation ADP induced in 40 ± 3% and 35 ± 1%, respectively, as compared to negative control (*P* < 0.05) (Figure 4). 

### 3.8. Adenosine Content in Ripe Tomato Fruits and Its Processing by-Products

**Figure 5:**
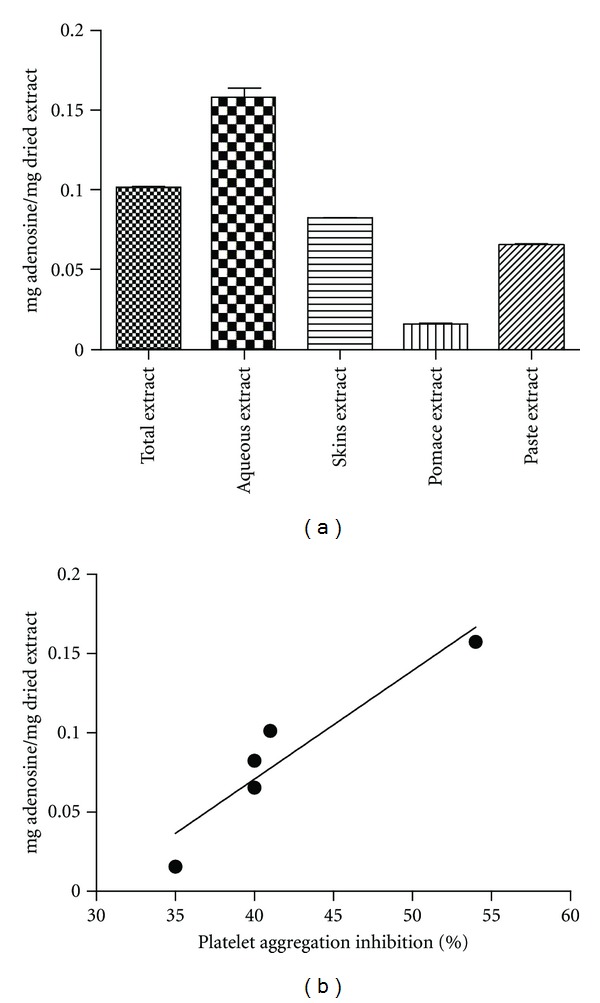
Quantitative analysis by HPLC of adenosine in ripe tomato fruits and their processing by-products. (a) Adenosine content expressed in mg adenosine/mg dried extract and (b) correlation coefficient and related significance between adenosine content and platelet inhibition (*r* = 0.94, *P* < 0.05). Total and aqueous extracts are obtained from tomato pulp. All the analyses were repeated at least twice starting from independent dried extracts.

The adenosine content in ripe tomato fruits and its processing by-products are presented in Figure 5. Such results were calculated from an adenosine standard linear regression with a correlation coefficient of *r* = 0.994. The pulp extracts (total and aqueous extracts) showed the highest content of adenosine, while tomato pomace showed the lowest amount. Finally, adenosine content in each extract was positively correlated with the inhibition of platelet function measured by platelet antiaggregant activity (*r* = 0.94, *P* < 0.05) (Figure 5).

## 4. Discussion

Epidemiological studies have provided evidence of a protective role of healthy diets in the prevention of cardiovascular disease and cancer [28]. Among the protective activities reported for tomato, its antiplatelet activity has been associated with a decrease in the prevalence of CVD [29].

It was observed that tomatoes exert *in vitro* [30] and *in vivo* [31] antiplatelet activity through the inhibition of platelet aggregation induced by ADP and collagen, as confirmed by our research group [29]. Recently, aqueous and methanol total extracts of red tomato were found to be thermally stable in the temperature range of 20 to 100°C and both acid and alkali did not affect inhibition of platelet aggregation induced by ADP. The presence of lycopene in the fraction extracted with water and methanol, respectively, were extracted showing the highest antiplatelet activity [32].

For extraction and fractioning stages, were fully ripe tomato fruits used showing an optimal industrial quality based on the firmness and soluble solid content [33]. The liquid-liquid separation allowed aqueous extract to have a higher yield than ethyl acetate and petroleum ether extracts. This may occur due to the high moisture content shown in tomato pulp (28).

Thus, in this study, liquid-liquid separation allowed concentrating antiplatelet activity in the aqueous extract, discarding the presence of compound in ethyl acetate (e.g., carotenoids) and petroleum ether extracts (e.g., triterpenoids and fatty acids) [26]. Such absence of these compounds is related to the slight antioxidant activity shown by the aqueous extract, which possibly allows us to establish that in such extracts a correlation between antioxidant and antiplatelet activities does not exist [19]. Conventional chromatography techniques (Sephadex LH-20, TLC preparative, and HPLC) used in the bioassay-guided allowed obtaining extract, fraction and bioactive compound from tomatoes with antiplatelet activity.

In the present study, besides the known platelet antiaggregant activity, it was demonstrated that aqueous extract inhibits platelet function completely: platelet adhesion, secretion, and aggregation.

Adenosine at a low concentration showed a potent antiplatelet activity through the inhibition of platelet aggregation and secretion; it also displayed a net lag time in the platelet aggregation assay induced by arachidonic acid, reaching the maximum aggregation rate of >80% at 360 s (data not shown). Moreover it presented inhibition of platelet thrombus formation under flow conditions. Adenosine, which can reach circulating blood from the heart, endothelium, and other tissues, is an endogenous antiaggregating substance that influences the function of circulating platelets by increasing cAMP and cGMP levels [34].

The quantitative HPLC analysis revealed that significant amounts of adenosine were contained in ripe tomato fruits and its processing by-products. Then liquid-liquid separation, the major content of adenosine in aqueous extract, is due to the affinity characteristics of this compound by polar extracts. On the other hand, the lowest adenosine content in tomato paste (rich in tomato pulp) may be due to the heat treatment, and to a lesser extent, because the sterilization stage during the paste-making process affects this compound negatively [35]. Tomato pomace is obtained as a by-product from processing tomatoes into fluid and pasty products such as tomato juice, sauce, and paste. Its content of adenosine is due to that it contains about 44% seeds and 56% of peels [36]. 

The present study confirms the antiplatelet activity of tomatoes; thus, based on the moisture rate and quantitative analysis of HPLC, 1 kg of fresh tomato contains about 30-times more adenosine than lycopene. So, tomato fruits and their by-products of processing have other bioactive compound, biologically complementing the activities of lycopene [37]. 

Finally, according to a correlation study, knowing the adenosine content in different extracts is possible to establish the degree of platelet aggregation inhibition. From these results, extracts/fractions of ripe tomato fruits and their by-products of processing may be referred to as functional food and functional ingredients containing a compound that inhibits platelet function with a potent preventive effect on thrombus formation, as those that occur in stroke.

## 5. Conclusion

Through bioassay guided on the basis of antiplatelet activity, was possible to isolate, identify, and determine a bioactive compound with a complementary biological activity to that of the lycopene. The antiplatelet activity is specific for adenosine, so that, according to its content, it is possible to establish the degree of such activity in the different types of extracts. Based on the present results, extracts of ripe tomato fruits and their processing by-products due to their adenosine content may be used as functional ingredients adding antiplatelet activities to processed foods which may be supportive in the primary prevention of CVD.

## Supplementary Material

Supplementary video 1: Adenosine Inhibit Collagen-Induced Platelet Thrombus Formation under Flow Conditions.
Supplementary video 2: Collagen-induced platelet thrombus formation under flow conditions.
Supplementary video 3: Aqueous Extract Inhibit Collagen-Induced Platelet Thrombus Formation under Flow Conditions.

## Figures and Tables

**Table 1:** Effect of extracts, fraction, and antiplatelet compound on platelet aggregation.

	ADP			
	Maximum aggregation (%)	Slope	Area under the curve	Lag time (s)
Total extract (1 mg/mL)	51 ± 8*	38 ± 2*	206 ± 10*	24 ± 3
Aqueous extract (1 mg/mL)	37 ± 2*	53 ± 7*	140 ± 56*	38 ± 5
Subfraction B (1 mg/mL)	1 ± 8*	6 ± 6*	2 ± 3*	>120*
Adenosine				
2.3 *μ*M	56 ± 2*	58 ± 2*	261 ± 9*	27 ± 1
4.6 *μ*M	40 ± 5*	44 ± 6*	201 ± 14*	34 ± 1
43 *μ*M	26 ± 8*	18 ± 12*	123 ± 15*	20 ± 1
457 *μ*M	21 ± 6*	28 ± 8*	98 ± 11*	53 ± 2*
Negative control	85 ± 2	104 ± 14	393 ± 21	30 ± 1

Values were presented in mean ± SD (*n* = 3). Band C corresponds to adenosine.

ADP 8 *μ*M. *P < 0.05 versus negative control (saline 0.9%).
